# Research on stem cell therapy for spinal cord injury: a bibliometric and visual analysis from 2018–2023

**DOI:** 10.3389/fgene.2024.1327216

**Published:** 2024-02-06

**Authors:** Ruxing Liu, Bo Peng, Jie Yuan, Jiahao Hu, Jianxin Yang, Nan Shan, Qichao Li, Bin Zhao, Chaojian Xu, Yongfeng Wang

**Affiliations:** ^1^ Department of Orthopedics, Second Hospital of Shanxi Medical University, Taiyuan, China; ^2^ Department of Shanxi Key Laboratory of Bone and Soft Tissue Injury Repair, Second Hospital of Shanxi Medical University, Taiyuan, China; ^3^ Department of Pain Management, Second Hospital of Shanxi Medical University, Taiyuan, China; ^4^ The Second Hospital of Shanxi Medical University, Taiyuan, China

**Keywords:** spinal cord injury, stem cells, tissue engineering, bibliometric analysis, exosome

## Abstract

**Objectives:** The aim of this study was to conduct a bibliometric analysis of the literature on stem cell treatment for spinal cord injury to gain an intuitive understanding of how the field is progressing, discover topics of interest, and determine what development trends are emerging in this field.

**Background:** Spinal cord injury and its complications often cause an enormous economic burden, and postinjury repair and treatment have always been challenging in clinical and scientific research. Stem cell therapy for spinal cord injury can prevent immune rejection and induce the release of neuroprotective and anti-inflammatory factors to reduce the production of stress-related proteins, reactive oxygen species, and inflammatory reactions.

**Methods:** We analyzed the number and quality of publications in the field of stem cell therapy in spinal cord injury between 2018.01.01 and 2023.06.30 in the core collection database of Web of Science. CiteSpace and VOSviewer were used to sort and summarize these studies by country, institution, authors' publications, and collaborative networks. In addition, the research topics of interest were identified and summarized.

**Results:** This study ultimately included 2,150 valid papers, with the number of publications showing a gradual upward trend. The country, institution, author and journal with the greatest number of publications and citations are China, the Chinese Academy of Sciences, Dai JW, and the International Journal of Molecular Sciences, respectively. The top three high-frequency keyword clusters were hereditary paraplegia, reactive astrocytes and tissue engineering.

**Conclusion:** With the help of visual analysis, we identified general trends and research topics of interest in the field of spinal cord injury over the last 5 years. Our findings suggest that stem cell transplantation for spinal cord injury and exosome therapy may be a focus of future research. This study provides a foundation for future research on stem cell therapy as well as clinical efforts in this field.

## Introduction

Spinal cord injury (SCI) is defined as compression of the spinal cord caused by traffic accidents, falls from heights, or sports injuries that results in corresponding clinical symptoms such as sensory and motor dysfunction, loss of vesicorectal sphincter function, and abnormal muscle tone below the level of the injury (Lenehan et al., 2012; Liu et al., 2023a). SCIs not only cause serious injuries to the patients but also impose an enormous financial burden on their families (Ke et al., 2023). Currently, although some progress has been made in the surgical and pharmacologic treatment of SCI, the effective repair of nerve defects is still a challenge in clinical treatment (Eaton et al., 2012). In the last 2 decades, the development of stem cells and regenerative medicine, as well as in-depth research on the pathophysiology of SCI, has provided new hope for its treatment (Fan et al., 2022; Zhang et al., 2022). Stem cells can proliferate and self-renew under certain conditions and can further differentiate into multiple functional cells (Ramotowski et al., 2019; Veneruso et al., 2019; Li et al., 2022). This new research direction has attracted the interest of many researchers, and a large amount of related literature has emerged. However, among the vast number of studies, identifying the articles with significant value; the countries, institutions and authors making important contributions to the field; and the current topics of interest and future directions of development is an important challenge. To date, many published reviews and expert opinions have attempted to shed light on the current state and frontiers of research on stem cell therapy for SCI (Tashiro et al., 2021; Zipser et al., 2022). However, these studies are relatively fragmented and subjective, insufficiently comprehensive or systematic, and less objective and quantitative, which is not conducive to obtaining an overall understanding of the field.

Bibliometrics, as a method of quantitatively summarizing multidimensional information, can be used to explore research trends in a particular field using visualization and web-related techniques, helping researchers grasp the current status of research and predict future research topics of interest in a short period (Meng et al., 2022; Cooper, 2015). The Web of Science (WoS) Core Collection is the most commonly used citation database in bibliometric analysis (Leng et al., 2013). The WoS can provide the following information about published studies: annual output, funding, authors, institutions, journals, and countries/territories (Cheng et al., 2022). VOSviewer and CiteSpace have become important tools for bibliometric analysis (Ma et al., 2023). They can be used to identify core researchers, countries and institutions in a certain research field and the mutual collaborations between them. Keywords can reflect global research trends and topics of interest, and they can generate visual pictures to express information more intuitively. Bibliometric methodology was used in a previous study to determine the status of stem cell research and its impact on the treatment of SCIs from 1999 to 2018, as well as future trends in the treatment of SCI (Guo et al., 2019). In this review, the stem cell types used to treat SCI included neural stem cells, Schwann cells, olfactory ensheathing cells, bone marrow mesenchymal stem cells and induced pluripotent stem cells. Therefore, an updated bibliometric analysis of the literature is necessary and meaningful.

Bibliometric analysis can be used to study the publications in a specific research field both quantitatively and qualitatively (Guo et al., 2019; Karova et al., 2019). With the use of visualization, we can demonstrate the structure, rules, distribution, and status of research, as well as topics of interest and development trends in this research field (Wang et al., 2023). We aimed to identify the current status and principal contributors to research in stem cell therapy for SCI from 2018–01-01 to 2023–06-30 and to predict future research trends in this field.

## Materials and methods

A search for publications related to stem cell therapy for SCI was conducted in the WoS Core Collection (SCI-EXPANDED and SSCI) on 1 July 2023. The search formula was as follows: [TS= (“spinal injury' OR “spinal cord injury" OR “spinal cord trauma" OR “spinal trauma")] AND [TS= (“stem cell" OR “stem cells")]. The search period was set from 2018.1.1–2023.06.30. The types of studies were limited to “Article” and “Review”.

First, a literature review was conducted using WoS to identify the annual outputs; authors; journals; institutions; countries and territories; and languages of the included studies. CiteSpace software (version 6.2. R4) and VOSviewer were used to identify the top authors, institutions, countries and collaborative networks. The CiteSpace parameters were set as follows: time slicing, 2018–2023; years per slice, 1; term source, all selections; node type, choose one at a time; selection criteria, top 50 objects; and pruning, pathfinder.

Bibliometric network construction and visualization were performed using VOSviewer. Comprehensive information about the authors, country/institutional collaboration, and co-occurrence of keywords was captured in VOSviewer graphs. For each node type, node size (i.e., frequency of occurrence or citation), internode centrality (labeled as a blue ring if ≥ 0.1, indicating a key node in the collaborative or cocitation network), explosiveness (red ring), and clustering of important nodes were included. A connecting line between two nodes indicated the existence of a collaborative relationship (between authors, institutions, or countries/regions) (Chen et al., 2012).

## Results

### Quantitative analysis of publication

**FIGURE 1:**
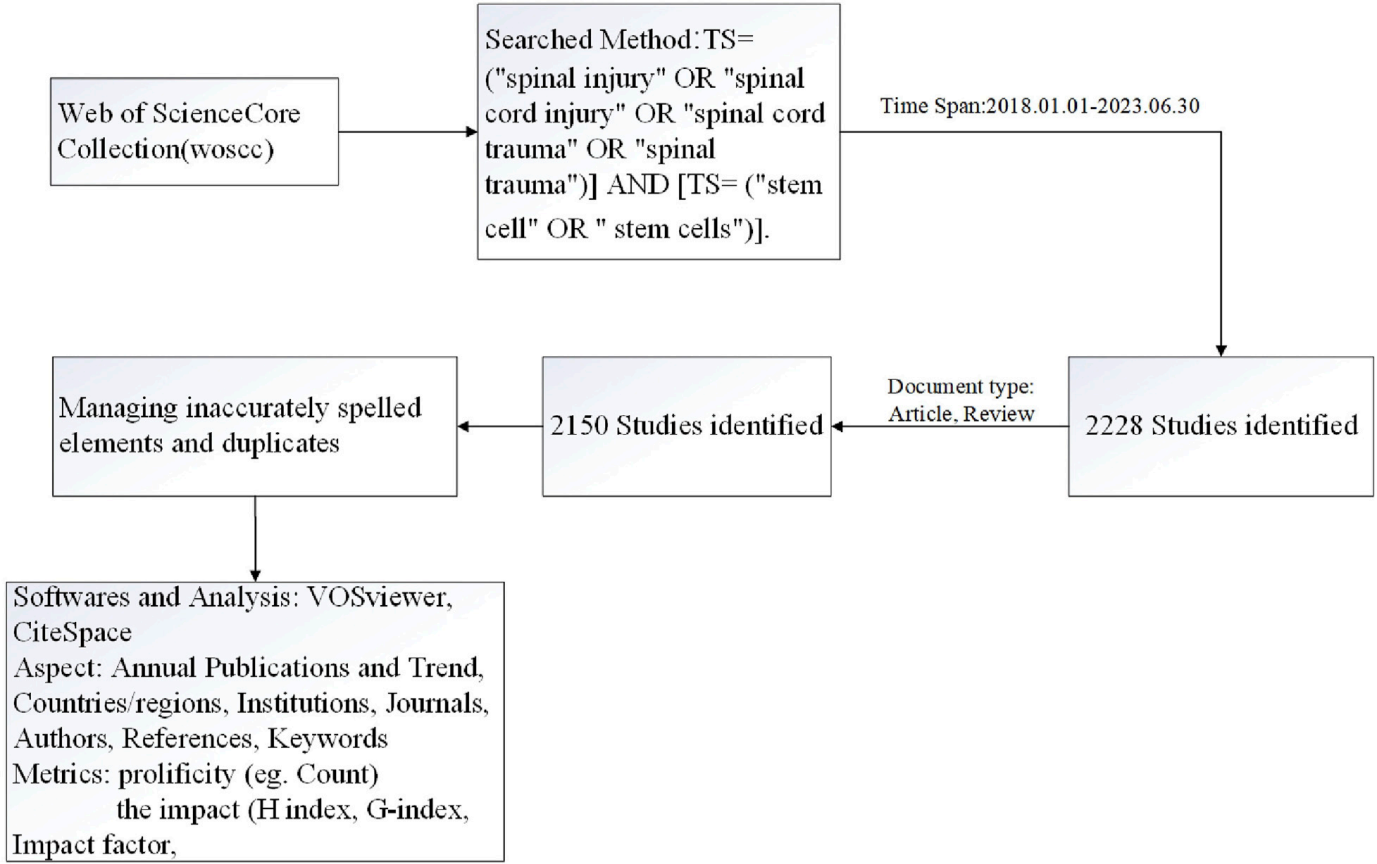
Article selection process for bibliometric analysis and systematic review.

**FIGURE 2:**
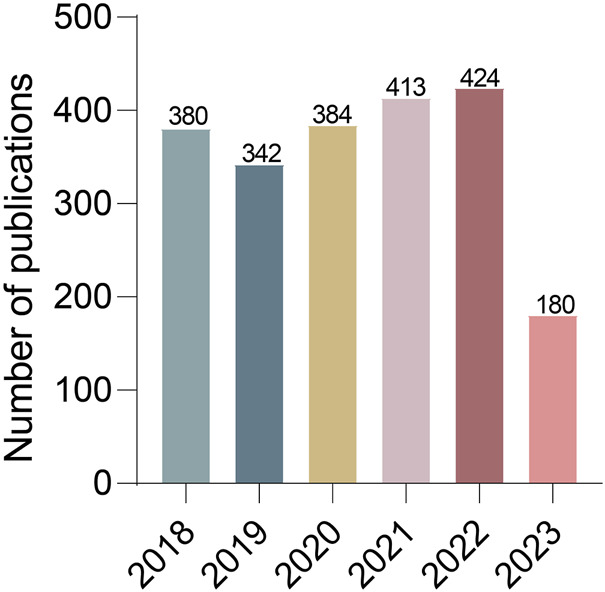
Distribution of publications on stem cell therapy for spinal cord injury from 2018 to 2023.

The search period was set from 2018.1.1–2023.06.30, and 2,228 literature records were retrieved. The types of literature were limited to “Article” and “Review”, and 2,150 English-language studies remained (Figure 1). These studies included 2,447 organizations from 71 countries, 88 authors, and 40,667 citations. Figure 2 shows that the number of articles on stem cell therapy for SCI has been steadily increasing from 2018 to 2023, indicating that this field has been increasingly studied by scholars and has become a topic of interest in SCI research. A total of 2,665 funding sources support research in this field. Table 1 lists the top 10 funding sources, of which the National Natural Science Foundation of China (NSFC), the National Institutes of Health (NIH, USA), and the United States Department of Health and Human Services are the top three funding sources.

**TABLE 1:** Top 10 funding sources for publications on Stem cells for spinal cord injury.

Ranking	Found source	Frequency	Ranking	Found source	Frequency
1	National Natural Science Foundation of China (NSFC)	560	6	National Key Research And Development Program Of China	58
2	National Institutes of Health (NIH, United States)	171	7	National Key R&D Program of China	57
3	United States Department of Health and Human Services	171	8	Chinese Academy of Sciences	49
4	Ministry of Education, Culture, Sports, Science, and Technology Japan (MEXT)	67	9	Grants In Aid for Scientific Research (Kakenhi)	47
5	Japan Society for the Promotion of Science	63	10	United Kingdom Research Innovation (UKRI)	47

### Journals analysis

From 2018 to 2023, a total of 589 journals published research related to stem cell therapy for SCI. Table 2 lists the 10 journals with the greatest number of published articles. These 10 journals published 20.046% of the total studies. The International Journal of Molecular Sciences is the most active journal in this field, followed by Neural Regeneration Research. Among journals with an impact factor (IF) greater than 3.00, Biomaterials has the largest IF (14.00). The average number of citations is more than 20 for articles published in some active professional journals, such as the International Journal of Molecular Sciences, Stem Cell Research Therapy, Frontiers in Cellular Neuroscience, Cell Transplantation, Biomaterials, and Stem Cells International.

**TABLE 2:** Top 10 productive journals that contributed publications on stem cells for spinal cord injury.

Ranking	Journal	Publications	Times cited	Times cited (per article)
1	International Journal of Molecular Sciences	68	1,390	20.44
2	Neural Regeneration Research	61	931	15.26
3	Stem Cell Research Therapy	53	1,218	22.98
4	Cells	51	760	14.9
5	Frontiers in Cellular Neuroscience	41	868	21.17
6	Biomaterials	35	1,257	35.91
7	Cell Transplantation	35	930	26.57
8	Scientific Reports	32	566	17.69
9	Journal of Neurotrauma	29	575	19.83
10	Stem Cells International	26	638	24.54

### Scientific collaboration network analysis

**FIGURE 3:**
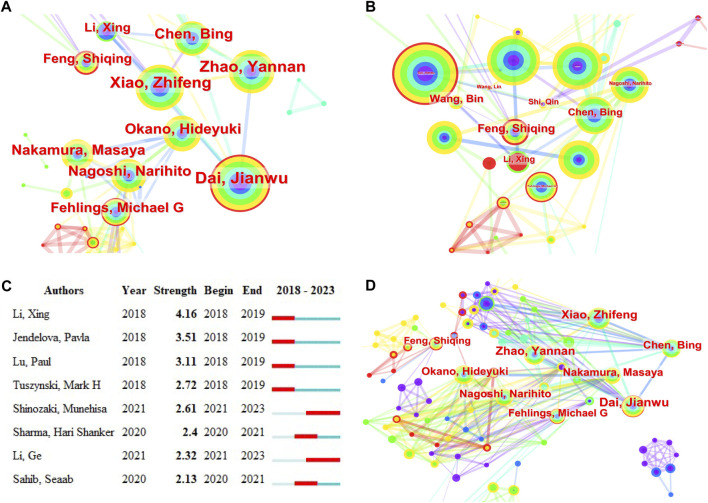
Co-occurrence analysis of authors. **(A)** The co-occurrence analysis of authors is represented by a network graph where the size of the circle indicates the number of publications, the more connections indicate more collaborations. **(B)** Top 10 authors in terms of centrality. **(C)** Authors emergence, red represents emergence, dark green represents the year in which the author appears, and light green represents the author does not appear **(D)** Collaboration network of some top authors.

Approximately 88 authors have indexed 2,150 articles on stem cell therapy for SCI. Table 3 lists the 10 authors with the highest number of publications and their institutions; these are specialized and active authors in this field. A co-occurrence map of authors was generated using CiteSpace. The h-index is a bibliometric parameter that simultaneously measures the quantity (number of documents) and quality (number of citations) of published papers and their increase over time. Betweenness centrality is used to measure the probability that a node is located on the shortest path between any other two points. The greater the betweenness centrality is, the more important the node. The betweenness centrality of the different authors is 0, indicating that the research in this field has not yet formed a widely connected core author network. Figure 3A shows the largest subnetwork of coauthors. The top three authors by co-occurrence count were Dai JW (Massoto et al., 2020), Wang Y (Zhang et al., 2019), and Li X (Huang et al., 2021), with h-indexes of 23, 16, and 19, respectively. The top three authors ranked by centrality were Chen Bing, Feng Shiqing, and Li Xing (Figure 3B). Figure 3C shows the authors with the strongest outbursts of publications in this field that increased rapidly over time; the top three authors were Li Xing, Jendelova Palva, and Lu Paul. The authors with the highest number of publications were broadly related to the others (Figure 3D).

**TABLE 3:** The top 10 active authors with the most publications from 2018 to 2023.

Rank	Author	Publications	Institution	Time cited	Time cited (per article)	H-index
1	Dai JW	46	Chinese Academy of Sciences	1,389	30.2	23
2	Wang Y	40	Uppsala University	1,107	27.58	16
3	Li X	39	Mudanjiang Medical University	1,201	30.79	19
4	Xiao ZF	39	Jilin Agricultural University	1,151	29.51	21
5	Zhao YN	37	Chinese Academy of Sciences	1,073	29	21
6	Chen B	33	Hangzhou Normal University	816	24.73	17
7	Okano H	31	Keio University	683	22.03	17
8	Nakamura M	30	Kitasato University	623	20.77	16
9	Nagoshi N	28	Keio University	684	24.43	16
10	Feng SQ	26	University of Toronto	726	27.92	13

### Co-occurrence analysis of keywords

**FIGURE 4:**
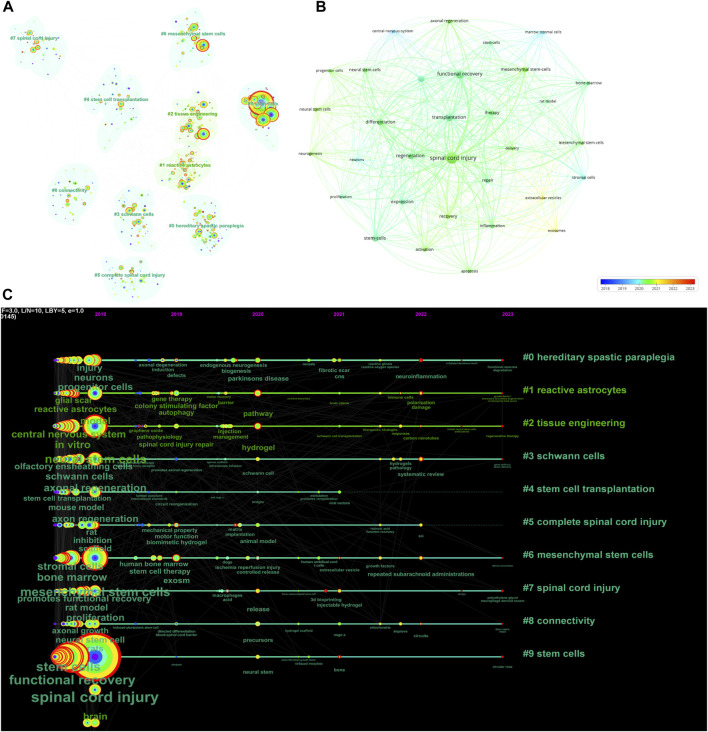
Mapping of keywords in studies on stem cells for spinal cord injury. **(A)** Keyword cluster analysis is represented by a network graph in which keywords from similar studies are grouped together; the same color represents a cluster. **(B)** Distribution of keywords according to average publication year (blue: earlier; red: later) generated by VOSviewer. **(C)** Keyword timeline visualization from 2018 to 2023 generated by CiteSpace.

Academic topics of interest and frontiers in a particular field are key topics in the academic community, reflecting the most current research issues and trends within that field. Using CiteSpace and VOSviewer, we analyzed the keywords through co-occurrence clustering to capture the research frontiers in this field. Keywords were classified into 10 categories: hereditary spastic paraplegia (Cluster 0), reactive astrocytes (Cluster 1), tissue engineering (Cluster 2), Schwann cells (Cluster 3), stem cell transplantation (Cluster 4), complete spinal cord injury (Cluster 5), mesenchymal stem cells (Cluster 6), spinal cord injury (Cluster 7), connectivity (Cluster 8), and stem cells (Cluster 9) (Figure 4A). Using VOSviewer, a network diagram was constructed based on the average year of publication (blue: earlier; red: later) (Figure 4B). We obtained a total of 43 keywords with a minimum of 66 occurrences. The six most common keywords were “spinal cord injury" (total link strength: 3,413), “functional recovery" (total link strength: 2082), “transplantation" (total link strength: 1846), “regeneration" (total link strength: 1,274), “differentiation" (total link strength: 1,211), and “stem cells" (total link strength: 1,009). Most of these keywords were published before 2020, while relatively new keywords appearing between 2021 and 2023 included “exosomes" and “extracellular vesicles".

**FIGURE 5:**
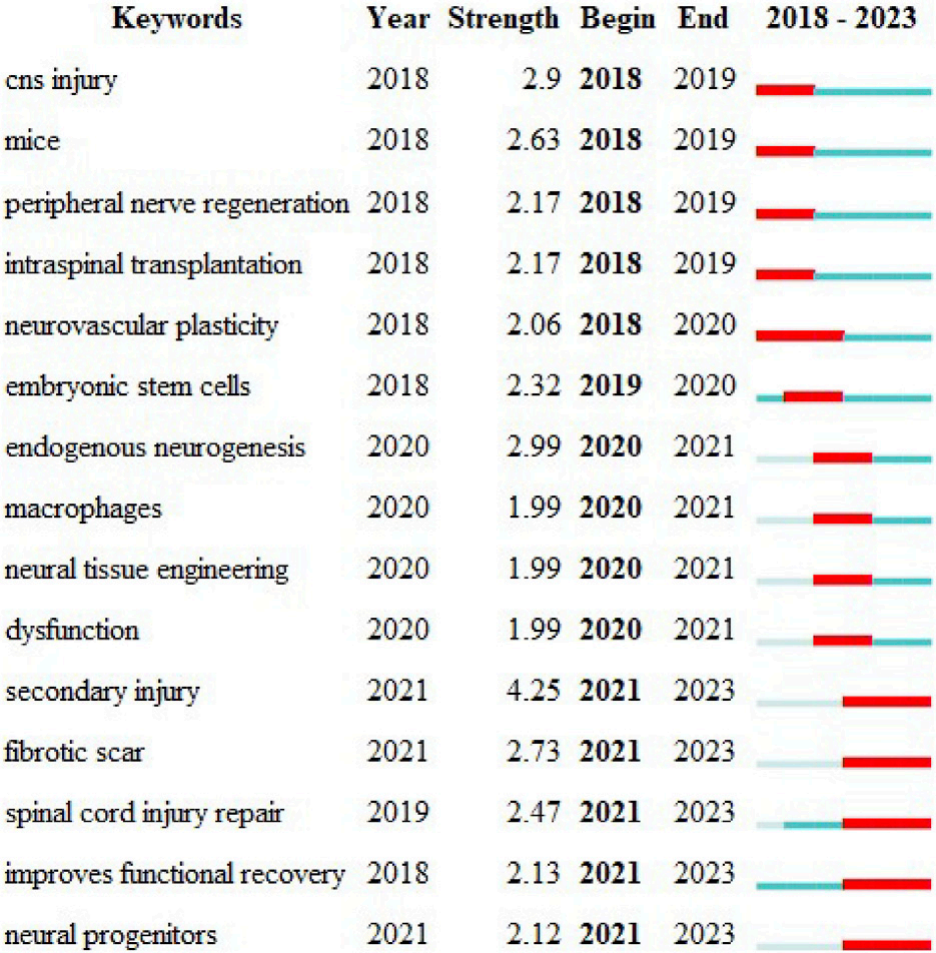
Keyword emergence: red represents emergence, dark green represents the year in which the keyword appeared, and light green represents that the keyword did not appear.

The temporal dynamic evolution of keyword clustering was visualized using CiteSpace (Figure 4C). The following ten major clusters were identified: “hereditary spastic paraplegia" (Cluster 0), “reactive astrocytes" (Cluster 1), “tissue engineering" (Cluster 2), “Schwann cells" (Cluster 3), “stem cell transplantation" (Cluster 4), “complete spinal cord injury" (Cluster 5), “mesenchymal stem cells" (Cluster 6), “spinal cord injury" (Cluster 7), “connectivity" (Cluster 8), and “stem cells" (Cluster 9). Clusters 0, 5 and 7 were the earliest research hotspots; Clusters 1, 3, 4, 6, 8 and 9 were midterm research hotspots; and Clusters 2, 4, 6, 7, 8, and 9 are current research hotspots. The CiteSpace algorithm was used to investigate the keyword bursts, revealing the 15 keywords with the strongest citation bursts. The keyword with the strongest citation burst was “secondary injury" (strength = 4.25), followed by “endogenous neurogenesis" (strength = 2.99) and “fibrotic scar". Interestingly, “secondary injury", “fibrotic scar", “spinal cord injury repair", “improves functional recovery", and “neural progenitors" had the highest number of citations in the recent bursts during 2021–2023, suggesting that these topics have the highest number of citations in recent studies; these topics are likely to be the next potential research topics of interest in the future (Figure 5).

### Distribution of countries/regions and institutions contributing to the field

**FIGURE 6:**
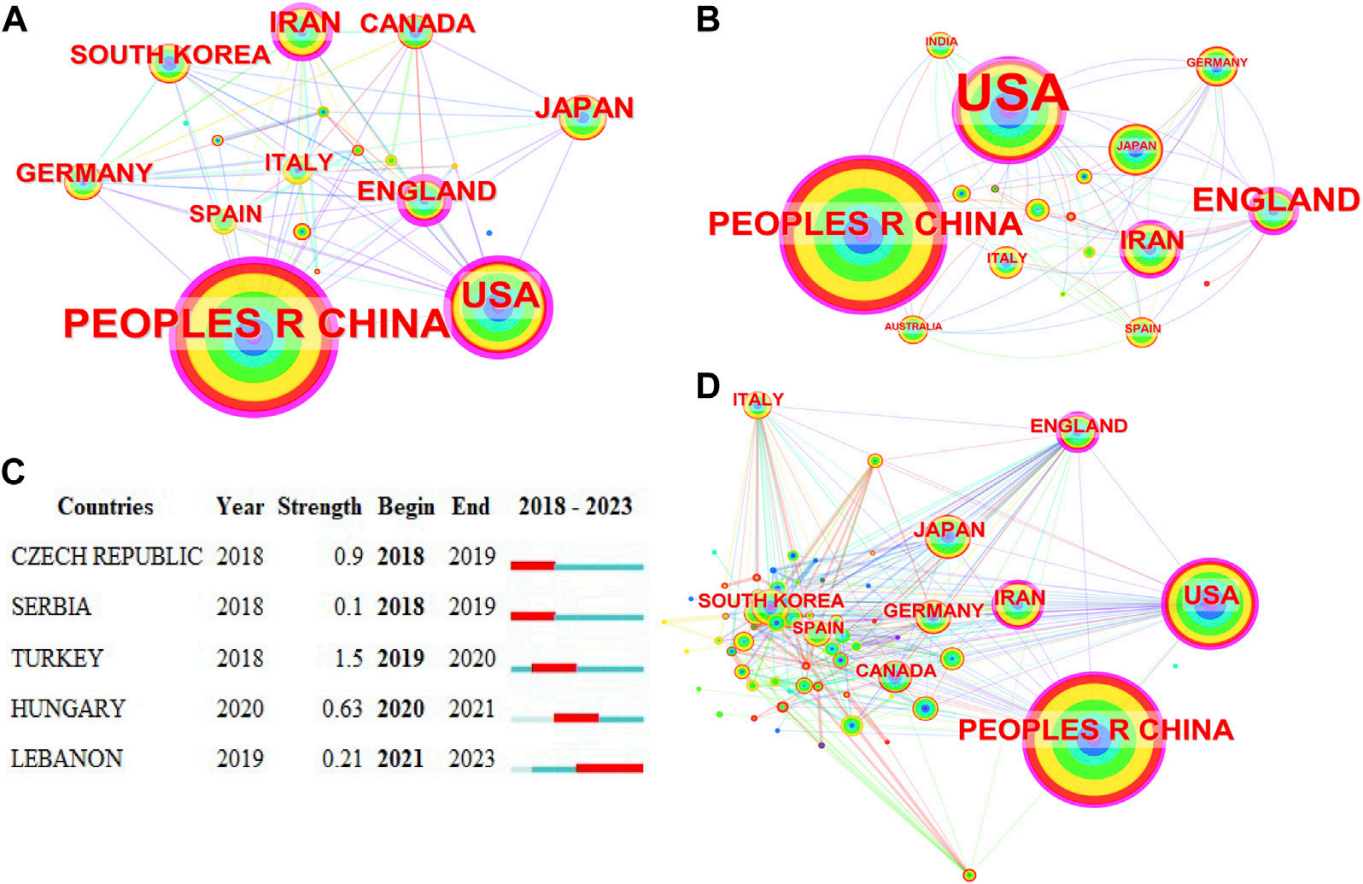
Co-occurrence analysis of countries/regions. **(A)** Top 10 countries in terms of co-occurrence counts. **(B)** Top 10 countries in terms of centrality. **(C)** Country emergence; red represents emergence, dark green represents the year in which the country appeared, and light green represents that the country did not appear. **(D)** In national cooperation, the larger the circle is, the more papers the country publishes and the more connections and cooperation between the country and other countries. The darker the color of the circle is, the greater the intensity of cooperation, and the greater the density around the ring is, the more the country is in the center of the research.

A total of 70 countries/regions have published studies in this field. The collaboration network diagram is shown in Figure 6. Table 4 lists the top 10 countries in terms of number of publications, collaborations, and centrality. The top three countries/regions by co-occurrence are the People’s Republic of China, the U.S., and Japan. The top three countries by centrality are the People’s Republic of China, the U.S., and England. There is a certain degree of cooperation between countries. The countries with the largest increase in the number of publications are shown in Figure 6C. The analysis of publications and centrality shows that China and the United States are the main research forces in this field.

**TABLE 4:** Top 10 most productive countries/regions that contributed publications on stem cells for spinal cord injury.

Rank	Country/region	Article count	Percentage (%)N/2,150)	Citation	Average citation
1	People’s Republic of China	918	42.698	17,484	19.05
2	United States	456	21.209	10,919	23.95
3	Japan	150	6.977	3,220	21.47
4	Iran	138	6.419	2,129	15.43
5	England	106	4.930	2,686	25.34
6	Germany	89	4.140	1,779	19.99
7	Canada	80	3.721	2,132	26.65
8	South Korea	80	3.721	1,025	12.81
9	Italy	67	3.116	1,665	24.85
10	Spain	61	2.837	1,144	18.75

**FIGURE 7:**
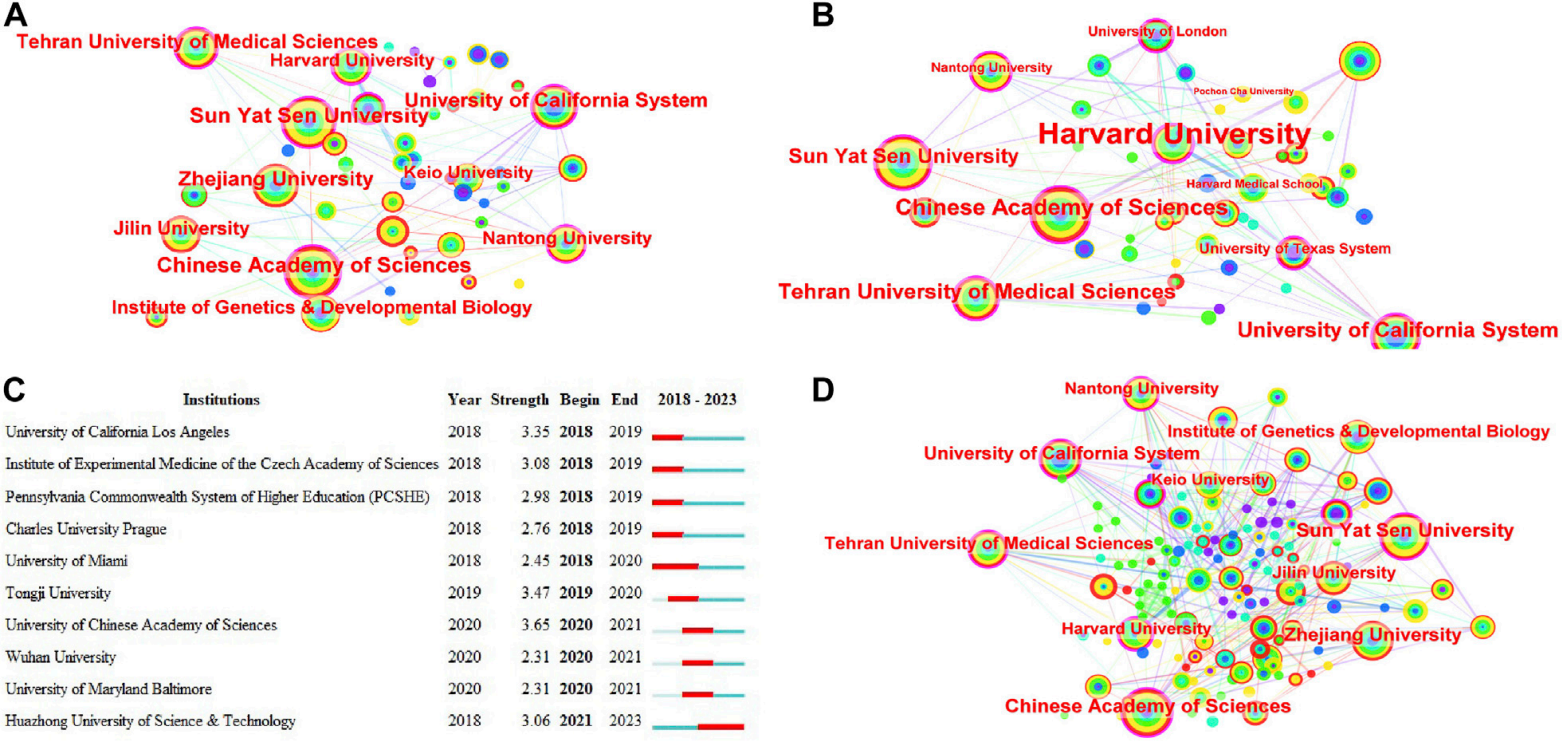
Co-occurrence analysis of institutions. **(A)** Top 10 institutions in terms of co-occurrence counts. **(B)** Top 10 institutions in terms of centrality. **(C)** Institution emergence: red represents emergence, dark green represents the year in which the institution appeared, and light green represents that the institution did not appear. **(D)** Cooperation network of some top institutions and related institutions that cooperate with Nantong University.

A total of 2,447 research institutions have published in this field over the past 5 years, and the largest subnetwork of interinstitutional collaborations is shown in Figure 7A. Table 5 lists the top 10 institutions in terms of publications and number of cooccurrences. The top three institutions according to the number of cooccurrences were the Chinese Academy of Sciences, Sun Yat-sen University, and the University of California System. The top three institutions according to centrality are Harvard University, Tehran University of Medical Sciences, and the Chinese Academy of Sciences. Burst monitoring identified 10 institutions, as shown in Figure 7C. The top three institutions were the University of the Chinese Academy of Sciences, with a burst from 2020 to 2021; Tongji University, with a burst from 2019 to 2020; and the University of California–Los Angeles, with a burst from 2018 to 2019.

**TABLE 5:** Top 10 institutions that contributed publications on stem cells for spinal cord injury.

Rank	Institution	Country	Article count	Percentage (%)(N/2,025)	Total citation	Average citation
1	Chinese Academy Of Sciences	China	87	4.047	2,693	30.95
2	Sun Yat Sen University	China	82	3.814	2,139	26.09
3	University Of California System	United States	74	3.442	2,496	33.73
4	Zhejiang University	China	66	3.070	1,660	25.15
5	Tehran University Of Medical Sciences	Iran	60	2.791	998	16.63
6	Harvard University	United States	49	2.279	559	11.41
7	Jilin University	China	48	2.233	700	14.58
8	Institute Of Genetics Developmental Biology Cas	China	46	2.140	1,389	30.2
9	Nantong University	China	44	2.047	729	16.57
10	Shanghai Jiao Tong University	China	39	1.814	1,298	33.28

Each top organization had extensive relationships with other institutions (Figure 7D). For example, important partners of the Chinese Academy of Sciences include the University of California System, Nantong University, Institute of Genetics and Developmental Biology, Jilin University, and Zhejiang University. Although Nantong University did not have many published articles, they have collaborated extensively with Sun Yat-sen University, Jiangsu University, Jilin University, Nanjing University, Nanjing Medical University, Stanford University, Chinese People’s Liberation Army General Hospital, National University of Singapore, The South China University of Technology and Shanghai Jiao Tong University, among others.

## Discussion

Currently, numerous studies address stem cell therapy for spinal cord injury, establishing a groundwork for its clinical translation (Zipser et al., 2022). However, these investigations exhibit fragmentation, subjective concentration, and limited comprehensiveness. Addressing the research focal points within this domain and predicting future developmental trajectories requires comprehensive assessment and elucidation.

Previous studies analyzed relevant literature from 1999 to 2018 and concluded that Cell Transplantation, Journal of Neurotrauma, Neural Regeneration Research and Experimental Neurology show more interested in this field. Okano H, University Toronto and USA were the top author, institution and country, respectively. Researchers and institutions from Canada, USA, Japan and China contributed the most in this field. Stem cell treatment of spinal cord injury is a promising field (Guo et al., 2019).

This paper is dedicated to exploring research prospects in a specific field by applying visualization techniques and network-related techniques. These methods can provide researchers with a concise overview of the current research status, predict future research hotspots, and lay a stronger foundation for the advancement of spinal cord injury treatment.

### Publication outputs

In this article, we used CiteSpace and VOSviewer software to conduct an intuitive and effective systematic analysis of relevant literature in the field of stem cell treatment for SCI. The visualization of the knowledge graph displays the cooperation status of countries/institutions, authors, cited journals, research topics of interest, *etc.*, revealing the research dynamics and cutting-edge trends in this field; this information has a positive guiding role in promoting the development of this field (Liu et al., 2023b). The number of publications represents, to a certain extent, the emphasis of the academic community on this research field (Shang et al., 2023). According to the publication trends, the number of articles on stem cell therapy for SCI has exhibited an annual upward trend. Therefore, stem cell treatment for SCI is attracting increasing amounts of attention. In the past 5 years, five journals have published more than 40 articles. These journals provide good platforms for research and academic exchanges on stem cell treatment in SCI. The International Journal of Molecular Sciences published the greatest number of articles, which indicates that this journal may become an important source of results for future research in stem cell treatment of SCI and should be given more attention.

### Scientific collaboration network analysis

Analyzing collaborations between authors, institutions, and countries can not only reveal the number of publications but also intuitively reflect their connections, development, and current status in the entire field, further revealing the structure and evolution of the discipline and providing a reference for the development of stem cell therapy for SCI. It can also help researchers utilize available resources with increased efficiency. The top 10 countries are located in North America (United States and Canada), Asia (China, Japan, South Korea, and Iran), and Europe (Germany, the United Kingdom, Italy, and Spain). China has the largest number of publications. Among the top 10 authors, 5 were from China: Dai JW, Li X, Xiao ZF, Zhao YN, and Chen B. The top institutions included the Chinese Academy of Sciences, Mudanjiang Medical University, Jilin Agricultural University, and Hangzhou Normal University. This may be because researchers and institutions in China publish more often. The three institutions with the most publications are the Chinese Academy of Sciences, Sun Yat-sen University and the University of California System. The top ten authors and their institutions are the main research forces in this field. The overall performance of Chinese institutions and authors is good, which may be related to the cooperation among institutions and authors and the large amount of funds invested by the National Natural Science Foundation of China in this field.

Research in this field has reached a certain level of maturity, and there has been wide and close cooperation among researchers worldwide, as indicated by the collaboration analysis. While some authors, institutions, and countries may have a small number of publications, they still have a significant number of partners. These could be emerging forces in this field and could become mainstays in the future. Combined with the number of publications in previous years, Okano Hideyuki, Nakamura Masaya and Fehlings Michael G, as the most published and cited authors in the field, focused on the molecular mechanisms of SCI and cell transplantation. For example, Prof. Okano Hideyuki of Keio University has published several important papers exploring the molecular mechanisms of SCI and the potential of induced pluripotent stem cells as a treatment (Okada et al., 2006; Sasaki et al., 2009; Tsuji et al., 2010; Nori et al., 2011). These studies have played an important role in the development of stem cell therapy for SCI. Xiao Zhifeng, Dai Jianwu, Zeng Yuanshan, and Sahib Seaab, as an emerging academic group in this field, are more concerned with the application of biomaterials in SCI (Xu et al., 2023). In summary, although stem cell therapy for SCI has received extensive attention from many countries, institutions, and authors, collaboration and communication between different research institutions and teams should be strengthened (Ma et al., 2021).

### Current research focus

Hotspots and frontiers in a field have always been popular topics in academia since they represent a specific field’s most concentrated research concerns and the latest trends in that field. An article’s keywords are usually highly condensed and focused summaries of its key points, and they are considered research hotspots if they occur frequently. A keyword co-occurrence analysis revealed that stem cell transplantation, tissue engineering, functional recovery, and exosome have been the most important keywords in this field over the past 5 years. The cutting edge topics included inflammation, neuroregeneration, exosomes, and glial scars. Recently, researchers have shown that suppressing inflammation and regulating glial scarring are important mechanisms for the repair of SCI by stem cells (Huang et al., 2017; Thompson et al., 2018; Karova et al., 2019). The use of the keyword “tissue engineering" also dramatically increased, indicating that translational medical research on stem cell treatment for SCI involves not only simple stem cells that can treat SCI but also methods of increasing the number of stem cells remaining in the injured area. Tissue engineering strategies can optimize the combination of seed cells, scaffold materials, and bioactive factors according to the specific requirements of spinal cord repair; significantly improve complex microenvironmental changes after SCI; and induce the differentiation of endogenous/exogenous neural stem cells into functional cells, thereby remodeling the microenvironment of neural regeneration, repairing the damaged neural network, and ultimately restoring motor function (Liu et al., 2020). Using biomaterials in conjunction with cytokines or stem cells can result in a reduction in the size of the injured area, a decrease in scar formation, enhancement of nerve regeneration, and restoration of motor function (Führmann et al., 2017; Zhao et al., 2017).

Increasing evidence suggests that stem cells exert their therapeutic effects via paracrine mechanisms, primarily through the function of extracellular vesicles such as exosomes (Liu et al., 2021; Yuan et al., 2023). In addition to facilitating intercellular communication, exosomes transfer specific information from parental cells to recipient cells, which affects the recipient cell’s genotype or phenotype. By reducing the size of the spinal cord cavity, decreasing the rate of apoptosis, reducing inflammation, and stimulating angiogenesis and axonal regeneration, exosomes may promote functional recovery of the spinal cord (Huang et al., 2017; Sun et al., 2018; Yuan et al., 2019). The above functions of exosomes are dependent mainly on the fact that they contain a variety of proteins, DNA, mRNAs, and microRNAs (miRNAs) derived from parental cells (Kalluri and LeBleu, 2020). Compared to stem cells, exosomes have the advantages of longer *in vivo* survival, lower oncogenicity, and more efficient delivery (Huang et al., 2021). In addition to crossing the blood‒brain barrier and entering the central nervous system, exosomes are small vesicles that are unlikely to cause vascular obstruction after intravenous injection and can be used to deliver carefully selected miRNAs, short interfering RNAs (siRNAs), and drugs via genetic modification or via certain vectors (Zhang et al., 2019). Exosomes cannot replicate *in vivo* and rapidly disintegrate after drug release; therefore, treatment with exosomes is unlikely to cause tumor formation or malignant transformation (Kim et al., 2018). Oxidized nanoparticles have been introduced by exosomes and were then guided by a magnetic field to accurately target the desired region, greatly enhancing their targeting ability. A new direction for the treatment of SCI has been provided by the use of exosomes as therapeutic agents and drug carriers.

### Research frontiers

#### Tissue engineering

Stem cell transplantation alone cannot rebuild the complex structure and stability of the spinal cord, and biomaterials and neurotrophic factors cannot replace the neuronal loss that occurs during SCI; therefore, combined transplantation has become a common research direction. Tissue engineering strategies offer great promise for the treatment of SCI. During tissue engineering-mediated repair of SCI, biomaterial scaffolds play a significant role in providing physical bridging and guiding functions, as well as acting as carriers of seed cells or bioactive factors, to achieve successful outcomes. The joint application of tissue engineering scaffolds mainly involves combining different types of scaffolds, such as nanofiber scaffolds with hydrogels (Nguyen et al., 2018; Zaviskova et al., 2018), stem cells (Taguchi et al., 2019), or neurotrophic factors (Limongi et al., 2018). The histocompatibility of the tissue engineering scaffolds allows them to address defects in the formation of the cavities and scar tissues and promote the regeneration of axons by improving the microenvironment, and they have also achieved better efficacy in experiments (Massoto et al., 2020). The combination of stem cells, tissue-engineered scaffolds, and neurotrophic factors in cotransplantation and the optimal transplantation method and timing have been studied (Liu et al., 2019; Mafikandi et al., 2019; Kim et al., 2023). To increase the likelihood of successful tissue engineering, it is important to consider the regenerative environment in the patient’s body, as well as factors such as stiffness, electrical conductivity, and drug release from biomaterials. Many challenges remain in SCI repair research despite the use of biodegradable materials, such as hydrogels. Replicating the function of the natural extracellular matrix using biomaterial scaffolds and controlling the material preparation process, including the regulation of biomechanics, modulus, porous structure, micro/nanostructure, and effect on cell behavior, remain the focus of future research (Pan et al., 2021). In the future, SCI repair research will focus on biomaterials as the core of tissue engineering regenerative repair, combined with cells, bioactive factors, drugs, and other components aimed at regulating the regenerative microenvironment of SCI as well as rebuilding functional neuronal circuits.

### Mesenchymal stem cell-derived extracellular vesicles (Msc-Evs)

The treatment of SCI with Msc-Evs has attracted increasing amounts of attention in recent years. SCI leads to rupture of blood vessels around the spinal cord, hemorrhage, and axonal rupture of neuronal cells; this results in Walle degeneration, which exacerbates axonal necrosis and myelin sheath decomposition and thus inhibits axonal regeneration. After SCI, secondary injury often occurs. This increases the severity of the injury and plays an important role in the prognosis of the patient. MicroRNAs (miRNAs) in Msc-Evs can improve secondary injury and promote the repair and regeneration of neuronal cells (Pan et al., 2021).

### Msc-Evs inhibit the inflammatory response after spinal cord injury

A study found that (Zhang et al., 2021) the treatment of SCI rats and microglia with exosomes derived from bone marrow stem cells (BMSC-Exos) inhibited the level of proinflammatory factors and improved the motor function of the rats; the level of miRNA-181c was elevated and the phosphorylation of nuclear factor signaling was inhibited in the neuronal cells of the rats, suggesting that the BMSC-Exos can inhibit the cellular inflammatory response and protect damaged neuronal cells. When the central nervous system (CNS) is damaged, resting astrocytes transform into A1-type astrocytes, which promotes the apoptosis of neuronal cells and inhibits axonal growth. BMSC-Exos inhibit the transformation of A1-type astrocytes after SCI, thereby reducing the inflammatory response at the site of spinal cord injury and lowering the expression level of proinflammatory factors, such as TNF-α and C3; these results suggest that BMSC-Exos can inhibit the transformation of astrocytes to A1-type cells, thereby protecting neuronal cells (Liddelow and Barres, 2017; Liddelow et al., 2017).

### Msc-Evs inhibits apoptosis after SCI

Generally, neuronal cells cannot be regenerated after damage and apoptosis, which are caused by a series of reactions in the neurons after SCI. miRNAs are noncoding single-stranded RNA molecules encoded by endogenous genes that can participate in pathophysiological processes in organisms and play important roles in cell growth and development. miRNAs are closely involved in neuronal apoptosis, inflammation, oxidative stress and other responses after SCI (Redell et al., 2011). In another study (Li et al., 2020), Msc-Evs containing miRNA-544 were injected into a rat model of SCI, after which the levels of proinflammatory factors in the tissues were analyzed. The results showed that injecting miRNA-544-containing Msc-Evs into the SCI rat model significantly increased neuronal growth, reduced neuronal cell loss and apoptosis, and promoted the recovery of neurological function in rats.

### Msc-Evs promotes axonal growth of neuronal cells after SCI

Axonal growth of neuronal cells plays an important role in nerve repair and regeneration after SCI, and growth-associated protein (GAP-43) and neurofilament protein (NF-200) are important proteins in this process.

Previous studies have shown (Riazifar et al., 2017; Xu et al., 2019) that BMSC-Exos improve the sensory function of rats after SCI by acting on the signal transducer and activator of transcription 3 (STAT3)/GAP-43 pathway; the number of NF-200-positive cells and the expression of STAT3 and GAP-43 increased, suggesting that BMSC-Exos can be activated by STAT3 and GAP-43 in rat cells. Exos can improve the sensory function of rats and promote axonal regeneration through the STAT3/GAP-43 pathway, which in turn promotes the repair and regeneration of neuronal cells. Axonal growth after SCI is not limited to one form or affected by a single factor; therefore, different severities of SCI require different therapeutic regimens to promote axonal regeneration.

## Conclusion

Stem cells play an important role in the treatment of SCIs. In recent years, the number of papers published annually in this field has grown steadily and rapidly. China and the United States are the main countries conducting research in this field. Reactive astrocytes, exosomes, and tissue engineering are currently the main topics in this field. With the development of new therapeutic approaches through stem cell transplantation, exosome stabilization is a trend for future clinical applications.

### Limitations

Although the studies' topics were specified by the search criteria, we cannot guarantee that every document was entirely pertinent to the subject. In addition, our study did not take into account self-reference bias. Nonetheless, we think that this study can still be utilized to describe the overall situation and general trend in this field.
